# Inhibitors of Venezuelan Equine Encephalitis Virus Identified Based on Host Interaction Partners of Viral Non-Structural Protein 3

**DOI:** 10.3390/v13081533

**Published:** 2021-08-03

**Authors:** Allison Bakovic, Nishank Bhalla, Farhang Alem, Catherine Campbell, Weidong Zhou, Aarthi Narayanan

**Affiliations:** 1 National Center for Biodefense and Infectious Diseases, George Mason University, Manassas, VA 20110, USA; abakovic@gmu.edu (A.B.); nbhalla@gmu.edu (N.B.); falem@gmu.edu (F.A.); 2 DCE Consulting, Vienna, VA 22181, USA; ccampb16@gmu.edu; 3 School of Systems Biology, George Mason University, Manassas, VA 20110, USA; wzhou@gmu.edu; 4 American Type Culture Collection, Manassas, VA 20110, USA

**Keywords:** Venezuelan equine encephalitis virus, non-structural protein 3, host-proteome, small molecule inhibitors, mass spectrometry, viral proteome, eIF2S2, TFAP2A

## Abstract

Venezuelan equine encephalitis virus (VEEV) is a new world alphavirus and a category B select agent. Currently, no FDA-approved vaccines or therapeutics are available to treat VEEV exposure and resultant disease manifestations. The C-terminus of the VEEV non-structural protein 3 (nsP3) facilitates cell-specific and virus-specific host factor binding preferences among alphaviruses, thereby providing targets of interest when designing novel antiviral therapeutics. In this study, we utilized an overexpression construct encoding HA-tagged nsP3 to identify host proteins that interact with VEEV nsP3 by mass spectrometry. Bioinformatic analyses of the putative interactors identified 42 small molecules with the potential to inhibit the host interaction targets, and thus potentially inhibit VEEV. Three inhibitors, tomatidine, citalopram HBr, and Z-VEID-FMK, reduced replication of both the TC-83 strain and the Trinidad donkey (TrD) strain of VEEV by at least 10-fold in astrocytoma, astroglial, and microglial cells. Further, these inhibitors reduced replication of the related New World (NW) alphavirus Eastern equine encephalitis virus (EEEV) in multiple cell types, thus demonstrating broad-spectrum antiviral activity. Time-course assays revealed all three inhibitors reduced both infectious particle production and positive-sense RNA levels post-infection. Further evaluation of the putative host targets for the three inhibitors revealed an interaction of VEEV nsP3 with TFAP2A, but not eIF2S2. Mechanistic studies utilizing siRNA knockdowns demonstrated that eIF2S2, but not TFAP2A, supports both efficient TC-83 replication and genomic RNA synthesis, but not subgenomic RNA translation. Overall, this work reveals the composition of the VEEV nsP3 proteome and the potential to identify host-based, broad spectrum therapeutic approaches to treat new world alphavirus infections.

## 1. Introduction

Venezuelan equine encephalitis virus (VEEV) is a New World (NW) alphavirus that is naturally transmitted by mosquitoes and endemic in South America [1,2]. However, epizootic outbreaks in the United States causing febrile and neurological illnesses in equids and humans have occurred periodically [3]. NW alphavirus infection can progress to encephalitis with a <1% case fatality rate for VEEV, with persistence of long-term neurological sequelae in survivors [3,4]. VEEV is classified as a category B select pathogen by the National Institutes of Allergy and Infectious Diseases [5,6]. Currently, there are no FDA-approved vaccines or therapeutics available for the treatment of exposure to VEEV and resultant disease manifestations. The live-attenuated vaccine strain of VEEV TC-83 is administered to military and at-risk personnel only, due to high reactogenicity concerns [7].

Non-structural protein 3 (nsP3) is expressed from the positive-sense genomic RNA as a non-structural polyprotein precursor with nsP1, nsP2, and nsP4 that forms the viral replicase that synthesizes the negative-sense intermediate strand, the full-length genomic RNA, and the subgenomic RNA [8]. While enzymatic functions have been reported for nsP1, nsP2, and nsP4 in the replication complex, the role of nsP3 in the replication complex has yet to be fully understood, albeit implicated in a negative-sense RNA synthesis [9,10,11,12]. NsP3 is comprised of three domains: an N-terminal macrodomain, an alphavirus unique domain, and a C-terminal hypervariable domain (HVD) [9]. The HVD of alphavirus nsP3 is intrinsically disordered, hypervariable, and hyperphosphorylated on serine, threonine, and tyrosine residues [12,13,14,15,16]. The disordered structure of the HVD facilitates preferential interactions with host proteins that support efficient viral replication [9,13,16,17,18,19,20]. Additionally, the subcellular localization of nsP3 observed outside of viral replication complexes suggests a role for nsP3 separate from genomic replication during infection [14,21,22,23]. At least 92 host protein interactions with Old World (OW) alphavirus nsP3 and at least 32 host protein interactions with NW alphavirus nsP3 [9,24] have been proposed using a variety of experimental approaches with overlapping and unique interaction partners. An example of differential host factor preferences among alphaviruses is the interaction of nsP3 with members of the FXR and G3BP RNA-binding protein families which disrupt stress granule assembly while facilitating viral replication complex formation [16,23]. Old World alphavirus nsP3 contains FGDF motifs facilitating binding to G3BP protein members for replication complex assembly, whereas VEEV nsP3 lacks this motif and uses FXR protein members for replication complex formation [16,23]. However, eastern equine encephalitis virus (EEEV), another member of NW alphavirus, can interact with FXR or G3BP protein family members for viral propagation [13].

Small molecules that target viral proteins exhibit great antiviral potential, but also pose the challenge of generation of resistant and/or escape mutants. Small molecules that can target the host-interacting partners of viral proteins can potentially exert antiviral activity while reducing the potential for the development of resistance. Furthermore, host-based inhibitory strategies are also likely to be broad-spectrum in functionality [24]. Re-purposing small molecule inhibitors that are already FDA approved for alternate indications can also greatly accelerate the process of therapeutics development against such acutely infectious agents [25,26,27,28]. This drug repurposing approach has shown promise in identifying small molecules against alphaviruses. Structure-based-drug-design and in silico modeling detected 1.5 million compounds of interest against VEEV, with 23 displaying inhibition of the viral capsid protein interaction partner, Importin α/β [29,30]. Another study utilized a luciferase-reporter-expressing TC-83 virus to screen a library of 2747 FDA-approved inhibitors for antiviral activity [31]. A total of 20 compounds decreased luminescence levels following infection with Sorafenib significantly reducing VEEV, EEEV, SINV and CHIKV replication [31]. In VEEV infected cells, Sorafenib treatment resulted in a loss of phosphorylated eIF4E which correlated with suppressed capsid protein translation [31]. Collectively, these studies demonstrate the potential to develop broad-spectrum therapeutic strategies against alphaviruses by targeting host-interacting partners.

In this study, we demonstrate the in vitro antiviral activity of three small molecule inhibitors against alphaviruses. These inhibitors were identified by using mass spectrometry to identify interactions between VEEV nsP3 and host proteins. Treatment with inhibitors tomatidine, citalopram HBr, or Z-VEID-FMK decreased VEEV replication in human astrocytoma, astroglial, and microglial cells following infection with the attenuated (TC-83) or wild-type (TrD) viral strains. Furthermore, these inhibitors retained efficacy against the related NW alphavirus, EEEV. Reduction in positive-sense, but not negative-sense genomic RNA levels was observed upon treatment with these inhibitors in the context of VEEV infection of astrocytoma cells. TFAP2A knockdown using siRNA resulted in a modest reduction in TC-83 replication, whereas eIF2S2 knockdown reduced infectious particle production significantly. Collectively, our data demonstrate the relevance of host protein targets and the re-purposing of small molecule inhibitors for the development of medical countermeasures against alphaviruses.

## 2. Materials and Methods

### 2.1. Cell Culture and Viruses

Vero African green monkey kidney cells (ATCC, CCL-81), 293 T human embryonic kidney cells (ATCC, CRL-3216), U-87MG human astrocytoma cells (ATCC, HTB-14), HMC3 human microglial cells (ATCC, CRL-3304), and SVGp12 human astroglial cells (ATCC, CRL-8621) were obtained from the American Type Culture Collection (Manassas, VA, USA). Vero, 293 T and U-87MG cells were cultured in Dulbecco’s Modified Eagle’s Medium (DMEM, Quality Biological, 112-013-101CS, Gaithersburg, MD, USA) and supplemented with 4.5 g/L glucose, 2 mM l-glutamine (Fisher Sci, MT2005CI, Hampton, NH, USA), 10 ug/mL streptomycin (VWR, 45000-652, Radnor, PA, USA), and 5% heat-inactivated fetal bovine essence (FBE, VWR, 10805-184, Radnor, PA, USA) for Vero cells, 5% heat-inactivated fetal bovine serum (FBS, ThermoFisher, 10437028, Waltham, MA, USA) for 293T cells, or 10% FBS for U-87MG cells. HMC3 cells and SVGp12 cells were cultured in Eagle’s minimum essential medium (EMEM, VWR, 670086) and supplemented with 10% FBS and 10 ug/mL streptomycin and 10 U/mL penicillin. All cell lines were cultured at 37 °C and 5% CO_2_.

The live-attenuated VEEV strain, TC-83, was obtained from BEI Resources (NR-63) [32]. Wild-type virulent VEEV strain Trinidad donkey (TrD) was obtained from BEI Resources (NR-332). Wild-type EEEV strain (FL93-939) was obtained from BEI Resources (NR-41567). A plasmid encoding the full-length VEEV strain TC-83 genome was modified to express nano Luciferase as a cleavable component of the structural polyprotein (TaV-nLuc), as previously described [33,34]. The HA-tag sequence, 5′-TAC CCA TAC GAT GTT CCA GAT TAC GCT-3′, was placed at the C-terminus of the nsP3 protein between nucleotide location 5680 and 5681 in the TC-83 TaV-nLuc virus (NCBI Reference Sequence L01443 for VEEV TC-83) by site-directed mutagenesis performed by GenScript (Piscataway, NJ, USA) (Figure S2 in Supplementary Figure File). Mutation of the opal stop codon (UGA) in the TC-83 TaV-nLuc virus was performed by site-directed mutagenesis performed by GenScript to a strong, ochre stop codon (UAA) (Figure S1, in Supplementary Figure File). In vitro RNA transcription from plasmid DNA and virus cultivation was previously described [12]. The HA-tagged virus was validated by Western blot for HA-tag expression, confocal microscopy comparison to an HA-tagged nsP3 overexpression construct, and infectious titer comparison to wild-type TC-83 (Figure S2). The mutated stop codon virus was validated for abrogation of replication by evaluation of cellular stress as compared to TC-83. RNA was electroporated into Vero cells which were evaluated at 24 h post-electroporation for cytopathic effect (CPE) (Figure S1).

### 2.2. Transfection and Preparation of Cellular Lysates

A pCAGGS-backbone plasmid expressing N-terminus HA-tagged nsP3 of the VEEV ZPC738 strain was previously described [35]. An empty control plasmid, Invitrogen™ pcDNA™ 3.1^(+)^ (Fisher Sci, V79020), was included in all over-expression transfections as a negative control. Next, 293T cells were seeded at 4 × 10^6^ cells per T-175 culture flask 24 h prior to transfection. Then, 10 μg of plasmid DNA was transfected using Attractene transfection reagent (Qiagen, 301005, Germantown, MD, USA), according to the manufacturer’s instructions. Flasks were incubated at 37 °C and 5% CO_2_ for 24 h.

ON-TARGETplus SMARTpool siRNA against Transcription Factor AP2 alpha (TFAP2A, ID: L-006348-02-0005) and against Eukaryotic Translation Initiation Factor 2 subunit 2 (eIF2S2, ID: L-011549-00-0005) was obtained from Dharmacon. ON-TARGETplus non-targeting control pool (Dharmacon, D-001810-10-05) was used as a negative control at a concentration of 50 nM. Next, 293T cells were seeded at 5 × 10^5^ cells per well in 6-well plates 24 h prior to transfection. Then, 25 or 50 nM of indicated siRNA was transfected using DharmaFECT 1 Transfection Reagent (Dharmacon, T-2001-03) per manufacturer’s instructions. Mock-transfected cells, transfection with DharmaFECT reagent-only cells, and non-targeting control siRNA-treated cells were included as controls. Plates were incubated at 37 °C and 5% CO_2_ for 72 h and subsequently infected, as described, for 24 h.

For evaluation of viral genomic RNA translation, 293 T cells were either untreated or treated with 10 µM tomatidine diluted in culture media for 2 h. Five µg of the mutated stop codon TC-83 viral RNA was transfected using Attractene transfection reagent, according to the manufacturer’s instructions. Plates were incubated at 37 °C and 5% CO_2_ for 24 h.

For preparation of whole cell lysates from infected or transfected cells, media were removed, and cells were washed twice with phosphate buffered saline (PBS, VWR, L0119-0500). Cells were lysed with cell lysis buffer (CLB, Cell Signaling Technology, 9803, Danvers, MA, USA) and were supplemented with 1 mM phenylmethylsulfonyl fluoride (PMSF, Cell Signaling Technology, 8553S), 10 mM 1,4-Dithiothreitol (DTT) (Invitrogen, P2325), and phosphatase inhibitor cocktail (ThermoFisher, 78420). Lysates were vortexed every 5 min (min) for 20 min, centrifuged at 10,000 rpm for 10 min at 4 °C, and supernatants were collected. The total protein for each sample was quantified against a standard curve of bovine serum albumin (BSA, Fisher Scientific, BP1600, Hampton, NH, USA) using Bradford reagent (VWR, E530-1L) and measured using a Beckman Coulter DTX 880 multimode plate reader. For non-immunoprecipitate (IP) samples, a 1:1 volume of 2× Laemmli buffer supplemented with 100 mM DTT was added to lysate supernatants and boiled for 10 min.

### 2.3. Immunoprecipitation

Two milligrams of total protein were incubated overnight at 4 °C with rotation using 2 µg mouse IgG3 isotype control (Abcam, 18394, Cambridge, UK) or anti-HA tag antibody (Abcam, ab 18181). Magnetic Dynabeads coated with protein G (FisherSci, 10-003-D) were washed with citrate phosphate buffer pH 5.0 (50 mM Tris-HCL pH 7.5, 120 mM NaCl, 5 mM EDTA, 0.5% NP-40, 50 mM NaF, 0.2 mM Na_3_VO_4_, Protease cocktail tablet (Sigma-Aldrich, 11697498001, St. Louis, MO, USA) and added to the protein–antibody IP complexes. Rotation at room temperature proceeded for 40 min followed by 1x wash with TNE_100_ + 0.1% NP-40, 1× wash with TNE_50_ + 0.1% NP-40, and 2× wash with PBS. TNE buffers consisted of 100 mM Tris-HCl pH7.5 and 0.2 mM EDTA, with 100 mM NaCl for TNE_100_ or 50 mM NaCl for TNE_50_. For Western blot imaging, Laemmli buffer supplemented with 100 mM DTT was added and beads were boiled for 10 min. For samples subjected to mass spectrometry, the last PBS wash was removed and Dynabeads were stored at −80 °C until processed for analysis.

### 2.4. Western Blot

Boiled samples were resolved on 4–20% Tris-Glycine gels (ThermoFisher, XP04122), and transferred to polyvinyl difluoride (PVDF) membranes by wet transfer at 80 mA overnight at 4 °C. Membranes were blocked for 30 min at room temperature with 3% non-fat dried milk in Tris-buffered saline with 0.1% Tween-20 (TBS-T). Anti-HA tag antibody (Abcam, 18181) was diluted in 5% BSA in TBS-T at 1:1000 and incubated on membranes for 1 h at room temperature. Anti-TFAP2A antibody (Abcam, ab52222), anti-eIF2S2 (Abcam, ab184549), or anti-VEEV nsP2 antibody (KeraFast, EU015) were diluted in 5% BSA in TBS-T at 1:1000, 1:5000, or 1:1000, respectively, and incubated on individual membranes overnight at 4 °C. Membranes were washed thrice with TBS-T for 5 min and incubated with respective secondary HRP-conjugated antibodies (Fisher Sci, PI32460) diluted 1:10,000 in 3% non-fat dried milk in TBS-T at room temperature for 1 h. Membranes were washed twice for 5 min with TBS-T and twice for 5 min with TBS. Membranes were imaged with SuperSignal West Femto Maximum Sensitivity Substrate Kit (ThermoFisher, 34095) and Bio-Rad Molecular Imager ChemiDoc XRS system. For re-probing, membranes were stripped with mild stripping buffer containing 0.1 M glycine, 0.2 M NaCl, 0.1% Tween-20 at pH 2.5, and were blocked with 5% BSA in TBS-T for 30 min, incubated with HRP-conjugated anti-actin antibody (Abcam, ab49900), diluted to 1:10,000 in 5% BSA in TBS-T for 30 min, and re-imaged with the ChemiDoc XRS system (Biorad, Hercules, CA, USA). ImageJ software was used to quantitatively calculate band densities, signals were normalized to actin loading controls, and fold-change was calculated versus a mock-transfected control group. Full length blots are presented in Figure S1.

### 2.5. Liquid Chromatography-Mass Spectrometry

LC-MS/MS was performed in a similar manner as previously described [12,36]. Briefly, immunoprecipitated Dynabeads were treated to 8M urea and disulfide bonds were reduced with 1M DTT and alkylated with iodacetamide. Following a trypsin digestion (ThermoFisher, 25200056) for 4 h at 37 °C, peptides were eluted with ZipTip purification (Millipore, Z720070, Rockville, MD, USA), and resuspended in H_2_O with 0.1% formic acid. Orbitrap Fusion tandem MS/MS with nanospray reverse-phase liquid chromatography (ThermoFisher) was performed. Full-scan mass spectra were acquired in Orbitrap over 300 to 2000 *m*/*z* with a 30,000 resolution followed with MS^n^ scans by CID activation mode. The 3 most intense ions were selected for fragmentation using a 35 collision energy and activation at Q = 25 for 30 milliseconds. Dynamic exclusion and charge state rejection were enabled. Mass spectra were fitted against NCBI reference sequence AAB02516 or P27282 for the non-structural polyprotein sequence analysis with Sequest Bioworks software 3.3.1. (ThermoFisher).

### 2.6. Ingenuity Pathway Analysis

Ingenuity pathway analysis (IPA, Qiagen) was used to evaluate datasets obtained from mass spectrometry. Briefly, datasets obtained from IP-MS were evaluated for coverage of the nsP3 protein of interest, and host interactors common to the empty control vector samples were removed. The host proteins exclusive to nsP3 immunoprecipitation were inputted into IPA and evaluated for abundancy and repetition among the runs. IPA was used to generate a dataset identifying host interactors present in at least 2/4 or 2/3 MS runs, as well as chemicals and drugs that directly or indirectly targetted those host interactors.

### 2.7. Inhibitors

Tolcapone (S4021), etretinate (S4699), KU-60019 (S1570), tomatidine (S9430), and citalopram HBr (S4749) were obtained from Selleckchem. Z-VEID-FMK (ab142025) was obtained from Abcam. CCG-1423 (5233) was obtained from Tocris. SCH-23390 hydrochloride (HY-19545A) was obtained from MedChemExpress. N-Butylidenephthalide, (E) + (Z) (sc-279727) was obtained from Santa Cruz Biotechnology. Prior to use, all inhibitors were resuspended in dimethyl sulfoxide (DMSO), with the exception of n-Butylidenephthalide which was resuspended in ethanol.

### 2.8. Toxicity Screens

Cells were seeded in 96-well white plates 24 h prior to use at 1 × 10^4^ cells per well, or 5 × 10^4^ cells per well for 293T cells. Inhibitors were diluted to the indicated micromolar concentration (μM) in culture media. siRNA was diluted to the indicated nanomolar concentration (nM) using DharmaFECT Transfection Reagent and added to cells per manufacturer’s instructions. Diluted inhibitors or siRNA were added to the cells and plates incubated at 37 °C and 5% CO_2_. At 24 h post-treatment (hpt), culture media were removed from the wells and cell viability was measured using CellTiterGlo^®^ Luminescent Cell Viability Assay per manufacturer’s instructions (Promega, G7572, Madison, WI, USA). Luminescence was measured using a Beckman Coulter DTX 880 multimode plate reader. Cell viability for inhibitors was calculated as a percentage versus the DMSO vehicle control. Cell viability for siRNA was calculated as a percentage versus the mock-transfected control.

### 2.9. Infections

Cells were seeded in 96-well plates 24 h prior at 1 × 10^4^ cells per well. Inhibitors were diluted in culture media to the indicated concentrations, with the final concentration of DMSO ≤0.1%. Media were removed and cells were pretreated with media containing drug or 0.1% DMSO vehicle control for 2 h prior to infection. The virus was diluted in culture media to the indicated multiplicity of infection (MOI). At the time of infection, pretreatment was removed, and the viral inoculum was overlaid on cells for 1 h. After 1 h, virus inoculum was removed, cells were washed 1× with PBS, and media containing inhibitor was added to cells. For time of addition infections, fresh media were added after the removal of viral inoculum until the indicated time point post-infection, at which time the diluted inhibitor was added. A 2-h inhibitor pretreatment was included as an internal control. Cells were incubated at 37 °C and 5% CO_2_ for the indicated duration. At the indicated time point post-infection (hpi), viral supernatants were collected and/or cellular lysates were obtained, and all samples were stored at −80 °C prior to further use.

### 2.10. Plaque Assay

Vero cells were seeded in 12-well plates at a density of 2.5 × 10^5^ cells per well and incubated for 24 h. Infection supernatants were serially diluted 10-fold in culture media and overlaid on cells for 1 h. Wells were covered with Eagle’s minimum essential medium (without phenol red, FisherSci, 67-008-6), supplemented with 5% FBE, non-essential amino acids (FisherSci, 11-140-050), 1 mM sodium pyruvate (VWR, 45000-710), 2 mM l-glutamine, 20 U/mL penicillin, and 20 μg/mL streptomycin mixed 1:1 with 0.6% agarose (ThermoFisher, 16500100). At 48 hpi, cells were fixed with 10% formaldehyde for at least 1 h. The medium was removed and stained with a 1% crystal violet in 20% ethanol solution.

### 2.11. Luciferase and Bradford Protein Assay

Next, 293T cells were untreated or treated with 10 µM tomatidine diluted in culture media for 2 h. Cells were infected with the nLuc-TaV expressing TC-83 virus at MOI 0.1 as described and incubated for 24 h. Cellular lysates were obtained using 1× Passive Lysis Buffer (Promega, E1941) and the Nano-Glo Luciferase Assay System was used to quantify subgenomic translation by measuring luciferase activity per manufacturer’s instructions. An aliquot of cellular lysates were mixed with Bradford reagent (Bio-Rad, 5000006, California, USA), according to the manufacturer’s instructions, and a standard curve for total protein was established, as previously described [12]. Mock infected cells were used to establish limits of detection, and luminescence and absorbance were measured using a DTX 880 multimode plate reader (Beckman Coulter, Indianapolis, IN, USA). Luciferase levels were normalized to total protein (RLU/µg).

### 2.12. RNA Extraction

At the indicated time points, cells were washed with PBS and lysed with TRIzol Reagent (Invitrogen, Waltham, MA, USA). Intracellular RNA was extracted with a Direct-zol Miniprep RNA kit (Zymo Research, Irvine, CA, USA) according to the manufacturer’s instructions, and stored at −80 °C prior to further use.

### 2.13. Primer/Probes and cDNA

All primers and probes were designed and obtained from Integrated DNA Technologies (IDT) using the PrimerQuest Tool. Primer/probe sets targeting the capsid and nsP3 region of the viral genome, which have previously been described [12]. Briefly, the sets contained a double-quenched ZEN/IBFQ probe with a 6-FAM fluorescent dye attachment at the 5′ end. A 18S rRNA endogenous control primer/probe set was obtained from ThermoFisher (4333760T). Amplification of the negative-strand TC-83 RNA has previously been described [12]. cDNA specific to negative-strand TC-83 RNA was generated using a T7 promoter sequence tagged at the 5′ end of the negative-strand and the high-capacity cDNA reverse transcription kit (ThermoFisher, 4368814), according to the the manufacturer’s instructions.

### 2.14. RT-PCR

Semi-quantitative RT-PCR has been previously described for measuring levels of capsid, nsP3, and 18S [12] and used thermal cycling conditions adapted from Verso 1-step RT-qPCR kit (ThermoFisher, AB4101C), according to the the manufacturer’s instructions. Amplification of the negative-strand RNA has been previously described [12] and used thermal cycling conditions adapted from PowerUp SYBR Green (ThermoScientific, A25742) according to the the manufacturer’s instructions. All reactions were amplified using StepOnePlus™ Real Time PCR system and no template cDNA controls and mock infections were included for all analyses to establish the limits of detection. Quantification for qRT-PCR was calculated using a standard curve based on threshold cycle (*C*t) values. Semi-quantitative RT-PCR values were calculated using the ΔΔ*C*t method [37] with viral RNA normalized to 18S levels.

### 2.15. Statistical Analysis and Curve Fitting

All graphs represent the mean ± SD for all data obtained. Prism 7 (Graph Pad) was used for all statistical analyses and significance was determined using one-way ANOVA with Dunnett’s post test unless otherwise stated. Statistical significance values are indicated using asterisk for * *p* < 0.0332, ** *p* < 0.0021, *** *p*< 0.0002, **** *p* < 0.0001, and ns for not significant. Prism 7 was used for curve fitting and predicting CC_50_ values using nonlinear regression with least squares (ordinary) fit and the log(inhibitor) vs. response variable slope (four parameters) equation. R^2^ values for goodness of fit were acceptable >0.85.

## 3. Results

### 3.1. Mass Spectrometry Identifies VEEV nsP3 Host Interaction Partners as Part of the nsP3 Interactome

Alphavirus nsP3 appears to act as a hub for host protein interactions that support efficient viral replication and can drive cell specific preferences for host–protein interactions among different alphaviruses [9,23,24]. To date, 23 host–protein interactions with VEEV nsP3 have been identified [9,12,38,39]. A mass spectrometry approach was employed in order to an effort to expand the reported nsP3:host protein interactome and utilize those host protein targets to identify small molecule inhibitors with antiviral activity against alphaviruses.

**Figure 1:**
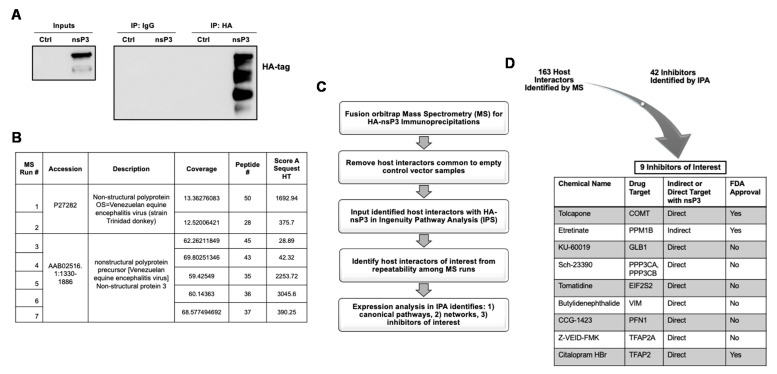
Mass spectrometry identified putative host interactors with VEEV nsP3 and potential antivirals. Then, 293T cells were transfected with a pcDNA 3.1^(+)^ empty control plasmid or a pCAGGS plasmid expressing N-terminus HA-tagged nsP3 of the ZPC738 VEEV strain. Next, 24 hpt, lysates were subjected to immunoprecipitation with anti-HA tag antibody or IgG3 isotype control antibody. Expression and immunoprecipitation of HA-nsP3 was confirmed by Western blot (**A**) and six independent immunoprecipitated samples were subjected to Fusion Orbitrap mass spectrometry (**B**), as described in Materials and Methods. Western blot and immunoprecipitations are representative of six independent experiments (*n* = 6). Mass spectra were fitted against the NCBI reference sequence AAB02516 or P27282 for the non-structural polyprotein of VEEV. (**C**) Schematic of data analysis by Ingenuity Pathway Analysis. Host proteins identified by mass spectrometry runs were analyzed for abundancy and identification of canonical pathways, networks, and inhibitors. (**D**) A list of small molecule inhibitors were identified using IPA for efficacy studies.

As the first step, 293 T cells were transfected with an overexpression plasmid encoding HA-tagged VEEV nsP3 at the N-terminus. Total protein lysates obtained from the transfected cells were used for immunoprecipitation (IP) using a HA antibody to enrich for nsP3-interacting host partners. Expression of nsP3 in transfected cells was confirmed by Western blot (Figure 1A, left panel), and a Western blot using IP samples (Figure 1A, right panel) confirmed the specificity of the HA-tag IP pulldown. HA-IP samples from both HA-nsP3 and the pcDNA empty control vector transfections (negative control) were subjected to liquid chromatography tandem mass spectrometry (LC-MS/MS). Spectra from MS runs 1–2 (Figure 1B) were fitted against the nonstructural polyprotein for the wild-type Trinidad donkey (TrD) strain. Spectra from MS runs 3–7 were fitted against the specific amino acids for nsP3 of the VEEV nonstructural polyprotein precursor (Figure 1B). Figure 1B displays the percentage of nsP3 protein covered by the number of peptides detected as well as the protein score (Score A SEQUEST HT), as measured by the individual scores of the peptides for a marker of protein identification. Total protein hits for the 7 MS runs were further evaluated to obtain interactors and inhibitors of interest (Figure 1C). Briefly, MS data were processed to remove host interactors identified in the HA-pcDNA IP from the HA-nsP3 IP samples in order to eliminate background hits and obtain host proteins that specifically interact with VEEV nsP3. These putative host interactors were evaluated using Ingenuity Pathways Analysis (IPA) to obtain a list of host proteins detected across multiple MS runs as well as inhibitors targeting the activity of these proteins. MS runs 1–4 were analyzed together and host proteins present in at least 2 of 4 runs were selected. Similarly, MS runs 5–7 were analyzed together and host proteins present in at least 2 of 3 runs were selected. The two sets of MS runs were analyzed independently in order to expand detection of potential host proteins that may be interacting with VEEV nsP3. In total, 160 putative host interactors with VEEV nsP3 (Supplementary Table S1) were identified along with 42 inhibitors targeting these host proteins as identified by IPA (Supplementary Table S2). The inhibitors prioritized by IPA were further analyzed in the context of published literature and the inhibitor list was narrowed to nine candidates for in vitro analyses of inhibitory potential (Figure 1D). These inhibitors were selected based on previous efficacy studies with related alphaviruses or other positive-sense RNA viruses, or if their predicted host target suggested a proviral role for replication.

### 3.2. Host-Based Inhibitors Display Strong Antiviral Activity against VEEV TC-83

**Figure 2:**
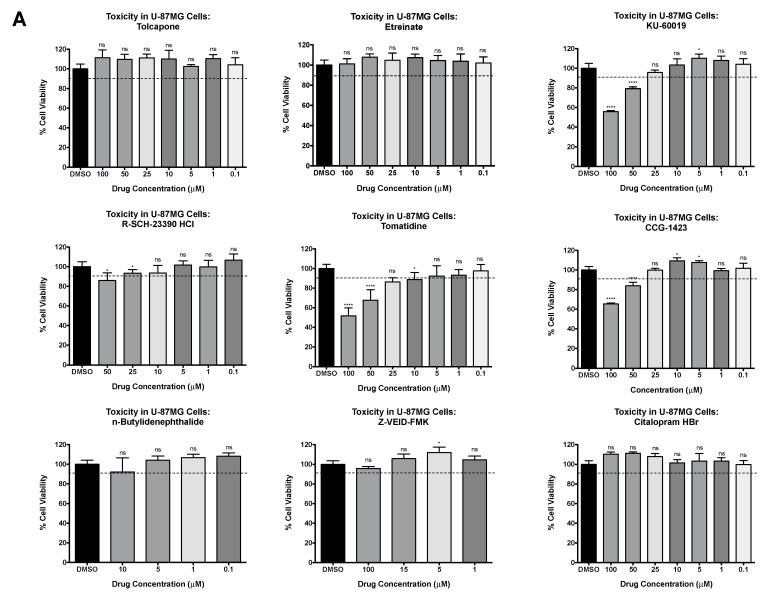

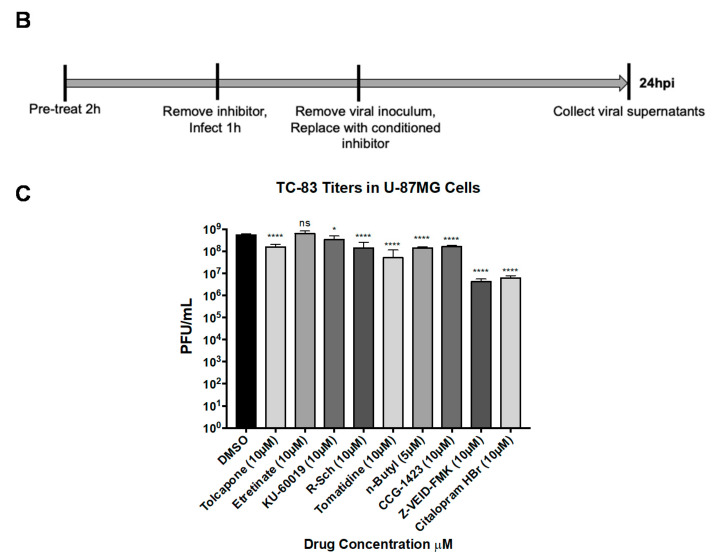
Efficacy of inhibitors against VEEV TC-83 in U-87MG astrocytes. (**A**) U-87MG cells were treated with small molecule inhibitors at varying concentrations (μM). Cell viability was measured at 24 hpt and calculated versus the DMSO vehicle control, as described in Materials and Methods. The dotted line represents the 90% cut-off point. (**B**) Schematic of experimental setup. (**C**) U-87MG cells were treated with a single, nontoxic concentration of inhibitor or 0.1% DMSO for 2 h. Cells were infected with TC-83 at MOI of 0.1 in triplicate for 1 h. Conditioned media containing inhibitor was replaced after removal of the virus. At 24 hpi, viral supernatants were collected and evaluated by plaque assay, as described in Materials and Methods. Graph represents data from two independent experiments performed in triplicate (*n* = 6). * *p* < 0.0332, **** *p* < 0.0001, ns, not significant.

The toxicity of selected inhibitors was assessed at multiple concentrations in U-87MG astrocytes [40,41]. Cell viability upon inhibitor treatment was evaluated against a 0.1% DMSO solvent control and cell survival of >90% was considered acceptable (Figure 2A). Notably, tolcapone, Eetretinate, n-Butylidenephthalide, Z-VEID-FMK, and citalopram HBr were nontoxic at all concentrations tested up to 100 μM. KU-60019, tomatidine, and CCG-1423 exhibited toxicity at concentrations ≥50 μM, and R-SCH-23390 HCl displayed toxicity at a 50 μM concentration. To determine the ability of these small molecules to reduce viral replication, U-87MG cells were pretreated with nontoxic concentrations for each of the nine inhibitors for 2 h and infected with VEEV TC-83 at MOI 0.1 (Figure 2B). Supernatants were collected at 24 hpi and evaluated for infectious titers by plaque assay (Figure 2C). Etretinate and KU-60019 failed to inhibit production of particle-forming units (PFU/mL) (ns, *p* < 0.0332, respectively). Tolcapone, R-SCH-23390, n-Butylidenephthalide, and CCG-1423 reduced TC-83 replication < 10-fold (*p* < 0.0001) compared to replication in DMSO-treated cells. In contrast, tomatidine reduced TC-83 replication by 11-fold (*p* < 0.0001), citalopram HBr by 87-fold (*p* < 0.0001), and Z-VEID-FMK by 128-fold (*p* < 0.0001) compared to replication in DMSO treated cells. The inhibitors displaying > 10-fold inhibition of VEEV TC-83 replication were chosen to further expand the in vitro efficacy studies.

### 3.3. Tomatidine, Citalopram HBr, and Z-VEID-FMK Display Efficacy against VEEV TC-83 Replication in Multiple Cell-Types

**Figure 3:**
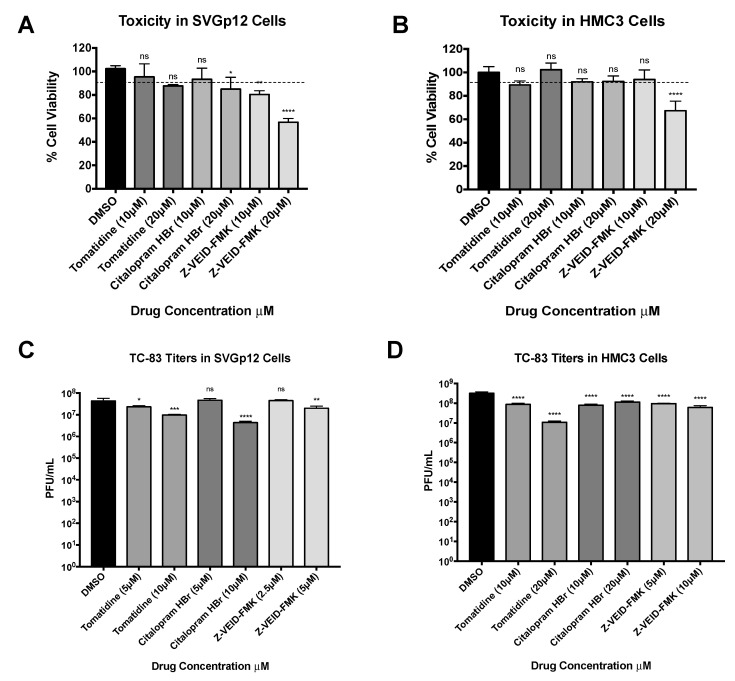
Tomatidine, citalopram HBr, and Z-VEID-FMK can inhibit TC-83 replication in multiple cell types. SVGp12 and HMC3 cells were treated with two concentrations of tomatidine, citalopram HBr, and Z-VEID-FMK. (**A**,**B**) Cell viability was measured at 24 hpt and calculated versus the DMSO vehicle control, as described in Materials and Methods. The dotted line represents the 90% cut-off point. (**C**,**D**) Cells were treated and infected in triplicate, as previously described (MOI 0.1), and viral supernatants were collected and evaluated by plaque assay, as described in Materials and Methods. Graph represents data from two independent experiments performed in triplicate (*n* = 6). * *p* < 0.0332, ** *p* < 0.0021, *** *p* < 0.0002, **** *p* < 0.0001, ns, not significant.

To evaluate cell type independence, the antiviral activity of tomatidine, citalopram HBr, and Z-VEID-FMK against VEEV was quantified in additional cells of the central nervous system (CNS). Human microglial cells (HMC3) and astroglial cells (SVGp12), with macrophage and fibroblast morphologies were chosen as these cells retain neuroinflammatory properties in vitro and are susceptible to VEEV infection [42]. Inhibitor associated toxicity was quantified in both cell lines by measuring cell viability against the DMSO solvent control (Figure 3A,B). Tomatidine and citalopram HBr were nontoxic at 10 μM (ns), whereas Z-VEID-FMK was toxic at 20 μM in HMC3 cells (*p* < 0.0001) and toxic at 10 μM in SVGp12 cells (*p* < 0.0021) (Figure 3A). The nontoxic concentration of each drug was subsequently used to test for inhibitory efficacy against TC-83 (Figure 3C,D). SVGp12 and HMC3 cells were pretreated with two concentrations of each drug and infected with TC-83 (MOI 0.1) and viral supernatants were evaluated for infectious titers at 24 hpi by plaque assay (Figure 3C,D). Tomatidine displayed a 4.5-fold decrease in replication levels at 10 μM (*p* < 0.0002), whereas citalopram HBr displayed a 10-fold decrease in replication levels (*p* < 0.0001) when compared to DMSO-treated SVGp12 cells (Figure 3C). All other treatment conditions displayed < two-fold reduction or no reduction in TC-83 replication levels in SVGp12 cells (*p* < 0.0332, ns). However, treatment of HMC3 cells with 10µM or 20 μM tomatidine (Figure 3D) reduced TC-83 titers by four-fold and 29-fold, respectively, in comparison with the DMSO control (*p* < 0.0001). Furthermore, 10 µM and 20 µM concentrations of citalopram HBr reduced TC-83 replication in HMC3 cells by four-fold and three-fold, respectively, in comparison to the DMSO control (*p* < 0.0001). Z-VEID-FMK treatment reduced TC-83 replication in HMC3 cells by three-fold and five-fold at 5 µM and 10 µM, respectively (*p* < 0.0001). Collectively, tomatidine and Z-VEID-FMK exhibited antiviral activity against VEEV TC-83 in HMC3 cells in a dose-dependent manner, whereas citalopram HBr displayed comparable inhibition at both tested concentrations.

### 3.4. In Vitro Selectivity Indexes (SI) of Tomatidine, Citalopram HBr, and Z-VEID-FMK

**Figure 4:**
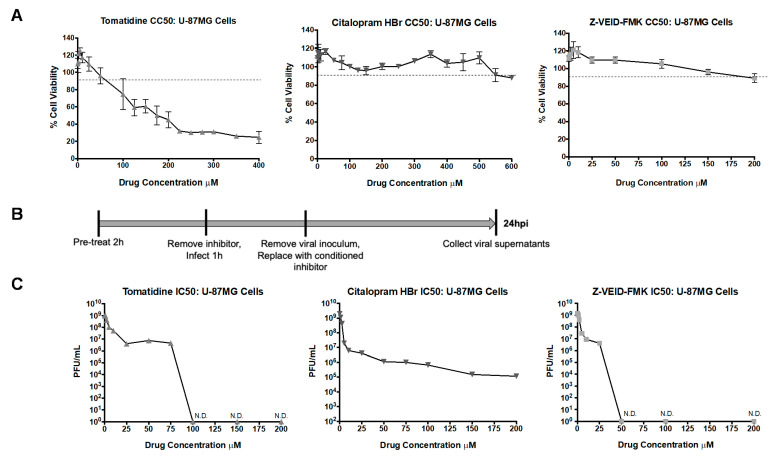
Selectivity indexes of tomatidine, citalopram HBr, and Z-VEID-FMK against VEEV TC-83. U-87MG cells were treated with varying micromolar concentrations of tomatidine, citalopram HBr, and Z-VEID-FMK. (**A**) cell viability was measured at 24 hpt and calculated versus the DMSO vehicle control, as described in Materials and Methods. The dotted line represents the 90% cut-off point. (**B**) Schematic of infection scheme (MOI 0.1). (**C**) Viral supernatants were collected at 24 hpi and evaluated by plaque assay, as described in Materials and Methods. Graph represents data from two independent experiments performed in triplicate (*n* = 6).

SI values are expressed as the ratio of the cytotoxic concentration, at which 50% cellular death occurs (CC_50_) to the effective concentration, and at which 50% reduction in viral load is observed following inhibitor treatment (EC_50_) [43]. U-87MG cells were used to determine SI values for tomatidine, citalopram HBr, and Z-VEID-FMK. Citalopram HBr is FDA-approved, exhibiting nanomolar potency while inhibiting apoptotic activity in vitro [44,45]. Z-VEID-FMK is a well-documented caspase-6 inhibitor demonstrating inhibition of fas-induced apoptosis at nanomolar concentrations [46,47]. Cell viability was measured for each inhibitor at 24 h post-treatment (Figure 4A). Tomatidine displayed a CC_50_ value of 175 μM while citalopram HBr and Z-VEID-FMK were nontoxic at most concentrations tested. To determine IC_50_ values, U-87MG cells were pretreated for 2 h, infected with TC-83 virus, and viral supernatants were evaluated by plaque assay at 24 hpi (Figure 4B). In addition, 50% inhibition of TC-83 replication was observed at 2.5 μM for tomatidine, 1 μM for citalopram HBr, and 0.5 μM for Z-VEID-FMK (Figure 4C). The resulting SI value for tomatidine was calculated as 70. Because a 50% cytotoxicity value was not determined for citalopram HBr and Z-VEID-FMK, a nonlinear regression curve analysis with least squared (ordinary) fit predicted CC_50_ values of 131,766 μM and 99,140 μM concentrations, respectively. Taken together, all three inhibitors, tomatidine, citalopram HBr, and Z-VEID-FMK exhibited high SI values in U-87MG cells in the context of VEEV infection.

### 3.5. Tomatidine, Citalopram HBr, and Z-VEID-FMK Inhibit Positive-Sense, but Not Negative-Sense Viral RNA Synthesis, and Post-Infection Treatment with Citalopram HBr, and Z-VEID-FMK Reduces Viral Replication

**Figure 5:**
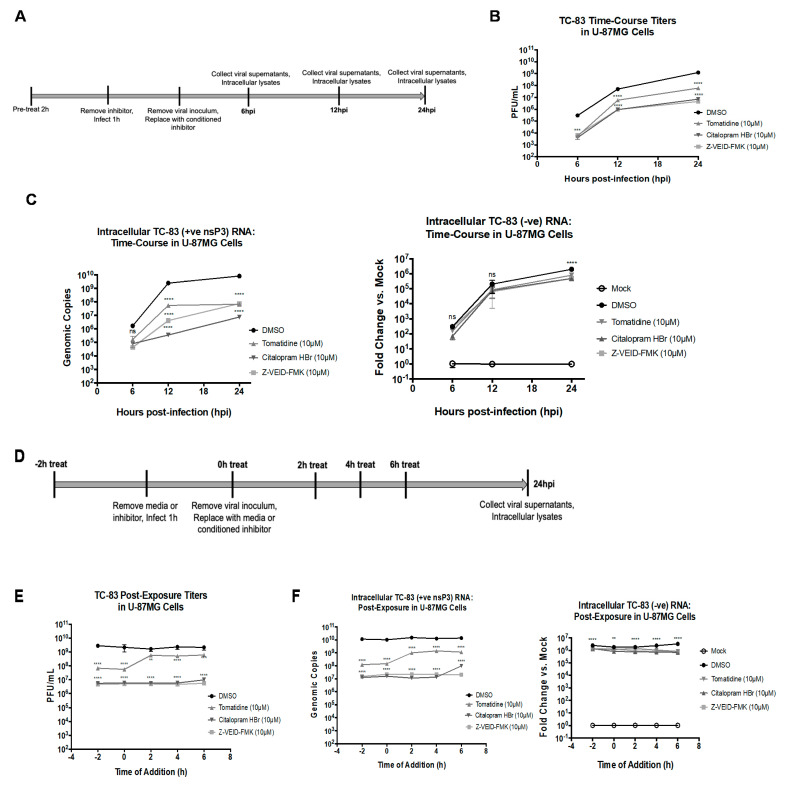
Time-course and post-exposure efficacy against VEEV TC-83. (**A**) U-87MG cells were pretreated for 2 h with nontoxic concentrations of tomatidine, citalopram HBr, and Z-VEID-FMK, subsequently infected with TC-83 at MOI 0.1 for 1 h, and conditioned media were replaced after the removal of viral inoculum. Viral supernatants and intracellular lysates were collected at 6, 12, and 24 hpi. (**D**) U-87MG cells were infected with TC-83 for 1 h at MOI 0.1. Nontoxic concentrations of tomatidine, citalopram HBr, and Z-VEID-FMK were added after the 1-h infection (0 h) or at 2, 4, 6 h post-infection. A 2-h pretreatment group with all drugs were included as a control. At 24 hpi, viral supernatants and intracellular lysates were collected. (**B**,**E**) Viral supernatants were evaluated by plaque assay, as described in Materials and Methods. (**C**,**F**) qRT-PCR to measure positive strand expression levels (nsP3) and RT-PCR to measure expression levels of negative strand was performed, as described in Materials and Methods. Graph represents data obtained from two independent experiments performed in triplicate (*n* = 6). ** *p* < 0.0021, *** *p* < 0.0002, **** *p* < 0.0001, ns, not significant.

To determine the steps of the VEEV replication cycle affected by tomatidine, citalopram HBr, or Z-VEID-FMK treatment, viral replication kinetics and levels of positive and negative-sense viral RNA strands in infected cells were quantified following pre-treatment or post-treatment with these inhibitors. First, the replication levels of TC-83 in U-87MG cells were measured at 6, 12, and 24 hpi after a 2-h pretreatment with each drug and subsequent infection at MOI 0.1 (Figure 5A). At each time point post-infection, viral supernatants were evaluated for infectious titers by plaque assay (Figure 5B) and intracellular lysates were obtained to quantify the positive- and negative- RNA strands by qRT-PCR and semi-quantitative RT-PCR, respectively (Figure 5C). At all time points, infectious particle production was inhibited for all three inhibitors tested (Figure 5B). Tomatidine reduced plaque formation as time points post-infection increased with a 55-fold, 9-fold, and 21-fold reduction observed at 6, 12, and 24 hpi, respectively, as compared to the DMSO control (Figure 5B). However, a more consistent and progressive inhibition of intracellular positive-sense RNA synthesis was observed at 10-fold, 45-fold, and 124-fold for 6, 12, and 24 hpi, respectively (Figure 5C). Citalopram HBr reduced plaque production by 72-fold, 54-fold, and 176-fold at 6, 12, and 24 hpi, respectively, as compared to the DMSO control (Figure 5B). The reduction in positive-sense RNA synthesis was inhibited variably upon citalopram HBr treatment, by 21-fold, ~10,000-fold, and 1000-fold at 6, 12, 24 hpi, respectively (Figure 2C). Z-VEID-FMK reduced plaque production as time points post-infection increased with a 44-fold, 53-fold, and 74-fold inhibition observed at 6, 12, 24 hpi, respectively, as compared to the DMSO control (Figure 5B), whereas, positive-sense RNA was reduced at 6, 12, and 24 hpi by 39-fold, 606-fold, and 110-fold, respectively, as compared to the DMSO control (Figure 5C). Collectively, citalopram HBr and Z-VEID-FMK reduce TC-83 plaque and positive-sense RNA production across all time points post-infection (Figure 5B)., In contrast to the large inhibition of positive-sense RNA levels observed, all three inhibitors reduced negative-sense RNA synthesis by <four-fold at all time points post-infection as compared to the DMSO control, as determined by semi-quantitative RT-PCR. Therefore, potential mechanisms of action for tomatidine, citalopram HBr, and Z-VEID-FMK involve inhibition of viral replication at a step ahead of and distinct from negative-strand RNA production.

Post-infection efficacy was assessed for these inhibitors by performing a time of addition assay in which U-87MG cells were treated with inhibitors at the time of infection (0 h) with TC-83 at MOI 0.1 or at 2, 4, or 6 h post-infection (Figure 5D). A 2-h pretreatment control group was included in the assay in a similar manner, as previously described (Figure 2B and Figure 4B). At 24 hpi, viral supernatants were assayed for infectious titers by plaque assay (Figure 5E) and intracellular lysates were assayed for positive- and negative-sense RNA levels (Figure 5F). Tomatidine inhibited particle production by 38-fold and positive-sense RNA synthesis by 71-fold when cells were treated at the time of infection as compared to the DMSO control (Figure 5D,E) (*p* < 0.0001). These inhibition levels were similar to the reduction observed in the 2-h pretreatment control where a reduction in particle production was 41-fold and positive-sense RNA synthesis was ~100-fold (*p* < 0.0001). Conversely, tomatidine inhibited TC-83 plaque production by <four-fold when cells were treated at 2, 4, or 6 h post-infection (*p* < 0.0021, *p* < 0.0001, *p* < 0.0002) (Figure 5E). A ~10-15-fold inhibition of positive-strand RNA levels was observed when tomatidine was added at 2, 4, or 6 h post-infection (*p* < 0.0001) (Figure 5F). Citalopram HBr and Z-VEID-FMK displayed consistent efficacy against TC-83 replication when administered at any time point post-infection, and inhibition was comparable to that observed in the context of pretreatment. Citalopram HBr inhibited plaque production by ~300-fold when cells were treated at the time of infection or at 2, 4, or 6 h post-infection, as compared to the 500-fold reduction observed following the 2 h pretreatment (*p* < 0.0001) (Figure 5E). Similarly, Z-VEID-FMK reduced plaque production by ~450-fold at the time of infection or at 2, 4, or 6 h post-infection as compared to the 600-fold inhibition observed following the 2 h pretreatment (*p* < 0.0001) (Figure 5E). Comparably, the reduction in positive-sense RNA synthesis was consistent upon treatment with Z-VEID-FMK as a ~550-fold reduction was observed when cells were treated at the time of infection or 2, 4, or 6 h post-infection as compared to the 750-fold reduction observed in the 2 h pretreatment (*p* < 0.0001) (Figure 5E). The time of addition study with citalopram HBr fluctuated in inhibition of positive-sense RNA synthesis where a 600-fold reduction was observed at the time of infection (*p* < 0.0001), a 1000-fold was observed upon treatment at 2 and 4 h post-infection (*p* < 0.0001), but only a 100-fold reduction was observed upon treatment at 6 h post-infection (*p* < 0.0001) (Figure 5E). Similar to the previous experiment (Figure 5C), negative-strand RNA synthesis was relatively unaffected by the inhibitor treatments post-infection at < four-fold reduction in comparison to the DMSO control for all time of addition time points for all inhibitor treatments (Figure 5D) (*p* < 0.0001). Collectively, citalopram HBr and Z-VEID-FMK exhibited a more robust inhibition against TC-83 replication when administered at post-exposure time points when compared to tomatidine. These data also demonstrate that tomatidine, citalopram HBr, and Z-VEID-FMK can inhibit positive-strand, but not negative-strand synthesis following treatment of cells both pre- and post-infection.

### 3.6. Tomatidine, Citalopram HBr, and Z-VEID-FMK Are Efficacious against the Virulent VEEV TrD Strain

**Figure 6:**
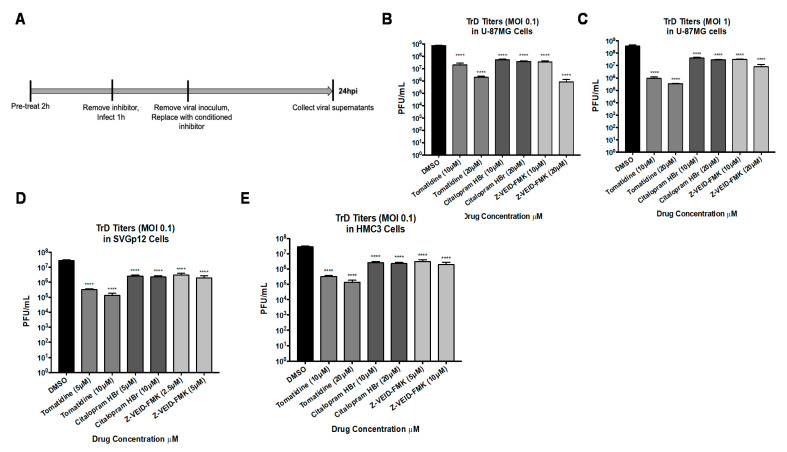
Tomatidine, citalopram HBr, and Z-VEID-FMK display efficacy against the wild-type VEEV Trinidad donkey strain in a cell-type independent manner. (**A**) Schematic of infection scheme. Cells were pretreated with two nontoxic concentrations of tomatidine, citalopram HBr, or Z-VEID-FMK for 2 h, subsequently infected with VEEV TrD at MOI 0.1 (**B**,**D**,**E**) or MOI 1 (**C**) for 1 h, and conditioned media were replaced after the removal of viral inoculum. Viral supernatants were evaluated by plaque assay, as described in Materials and Methods. (**B**,**C**) Efficacy in U-87MG cells at MOI 0.1 and 1, respectively. (**D**) Efficacy in SVGp12 cells. (**E**) Efficacy in HMC3 cells. Graphs are representative of two independent experiments performed in technical triplicates (*n* = 6). **** *p* < 0.0001.

The efficacy of tomatidine, citalopram HBr, or Z-VEID-FMK against the virulent TrD strain was determined in vitro (Figure 6A). To that end, U-87MG cells were pretreated with two nontoxic concentrations of tomatidine, citalopram HBr, and Z-VEID-FMK, and cells were subsequently infected with the TrD strain at MOI 0.1 (Figure 6B) or MOI 1 (Figure 6C), and viral supernatants were evaluated by plaque assay at 24 hpi. Tomatidine inhibited TrD replication at MOI 0.1 by 25-fold and 364-fold at 10 μM and 20 μM concentrations, respectively (*p* < 0.0001), in comparison to the DMSO control. Surprisingly, a greater level of inhibition of TrD replication was observed at MOI 1 for the same inhibitor concentrations at 400-fold and 1000-fold reductions of infectious particle production (*p* < 0.0001). Citalopram HBr reduced TrD replication at 10 μM and 20 μM by 14-fold and 19-fold at MOI 0.1 (*p* < 0.0001) in comparison to the DMSO control, whereas a lower level of inhibition was observed at MOI 1, where reduction in viral replication was 9-fold and 13-fold (*p* < 0.0001) when compared to replication in DMSO-treated cells. Z-VEID-FMK reduced TrD replication at 10 μM and 20 μM by 21-fold and 887-fold at MOI 0.1, whereas inhibition at MOI 1 was observed to be only a 12-fold and 45-fold reduction (*p* < 0.0001), when compared to DMSO treated cells (Figure 2C).

The cell type independence of these inhibitors against TrD was evaluated in both SVGp12 (Figure 6D) and HMC3 (Figure 6E) cells. Two concentrations of each inhibitor were used to pretreat cells which were subsequently infected with TrD at MOI 0.1 in the same manner, as previously described (Figure 6A). Viral supernatants were assayed for infectious titers by plaque assay at 24 hpi. Tomatidine inhibited TrD replication at 5 μM and 10 μM by 87-fold and 207-fold, respectively, in SVGp12 cells (*p* < 0.0001) in comparison to the DMSO control. Similarly, tomatidine reduced TrD replication at 10 μM and 20 μM in HMC3 cells by 87-fold and 207-fold, respectively (*p* < 0.0001). Similar to data from U-87MG cells, tomatidine displayed greater efficacy against the TrD strain than against TC-83 (Figure 2C). Citalopram HBr inhibited TrD replication by ~10-fold in both SVGp12 and HMC3 cells in a dose-independent manner at the indicated drug concentrations (*p* < 0.0001) and comparable to the inhibition observed with the TC-83 strain. Z-VEID-FMK inhibited TrD replication by 14-fold in both SVGp12 and HMC3 cells (*p* < 0.0001) and therefore, greater inhibition with this inhibitor was observed when compared to TC-83 (Figure 3C,D). Collectively, these inhibitors have demonstrated the ability to suppress TrD replication efficiently in multiple cell types.

### 3.7. Tomatidine, Citalopram HBr, and Z-VEID-FMK Can Inhibit EEEV Replication

**Figure 7:**
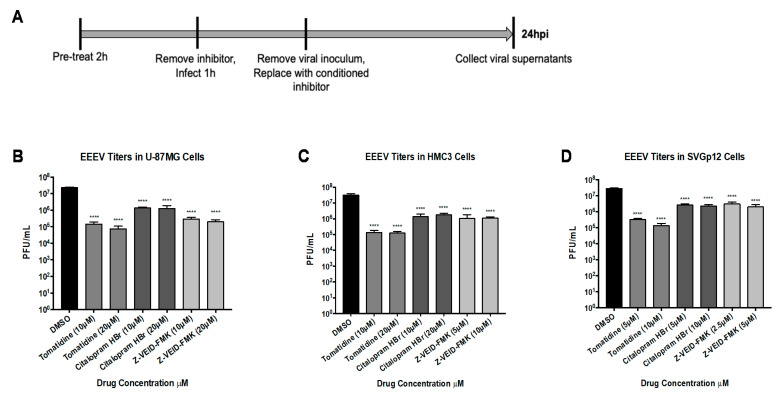
Tomatidine, citalopram HBr, and Z-VEID-FMK display efficacy against EEEV in a cell-type independent manner. (**A**) Schematic of infection scheme. Cells were pretreated with two nontoxic concentrations of tomatidine, citalopram HBr, or Z-VEID-FMK for 2 h, subsequently infected with EEEV at MOI 0.1 for 1 h, and conditioned media were replaced after the removal of viral inoculum. Viral supernatants were evaluated by plaque assay, as described in Materials and Methods. (**B**) Efficacy in U-87MG cells. (**C**) Efficacy in HMC3 cells. (**D**) Efficacy in SVGp12 cells. Graphs are representative of two independent experiments performed in technical triplicates (*n* = 6). **** *p* < 0.0001.

Tomatidine, citalopram HBr, and Z-VEID-FMK were next assessed for antiviral activity against the related NW alphavirus, EEEV. Cells were pretreated with two nontoxic concentrations of inhibitors, infected with EEEV at MOI 0.1, and viral supernatants were collected at 24 hpi and evaluated for infectious titers by plaque assays (Figure 7A). Tomatidine, tested at 10 and 20 µM concentrations, reduced EEEV replication by 157-fold and 314-fold, respectively, (*p* < 0.0001) in U-87MG cells (Figure 7B), 87-fold and 207-fold (*p* < 0.0001) in SVGp12 cells at 5 and 10µM (Figure 7C), and 238-fold and 259-fold (*p* < 0.0001) in HMC3 cells at 10 and 20 µM (Figure 7D) when compared to replication in DMSO treated cells. Citalopram HBr reduced EEEV replication by ~17-fold (*p* < 0.0001) in U-87MG cells (Figure 7B), ~11-fold (*p* < 0.0001) in SVGp12 cells (Figure 7C), and ~21-fold (*p* < 0.0001) in HMC3 cells (Figure 7D). Z-VEID-FMK reduced EEEV replication by ~100-fold (*p* < 0.0001) in U-87MG cells (Figure 7B), ~12-fold (*p* < 0.0001) in SVGp12 cells (Figure 7C), and ~30-fold (*p* < 0.0001) in HMC3 cells (Figure 7D). Taken together, these data support the use of tomatidine, citalopram HBr, and Z-VEID-FMK as potential pan-alphavirus inhibitors with tomatidine antiviral activity being most efficacious against both EEEV and VEEV in vitro.

### 3.8. eIF2S2 Supports Efficient TC-83 Replication and Genomic RNA Translation, and VEEV nsP3 Colocalizes with TFAP2A

**Figure 8:**
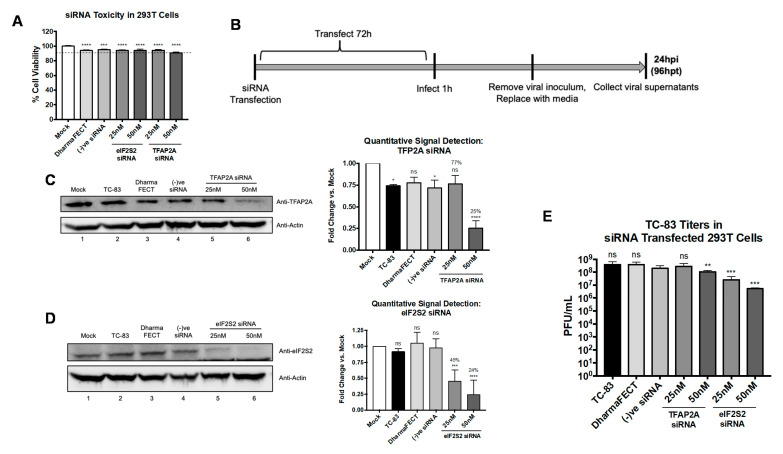
EIF2S2 supports a pro-viral role for VEEV TC-83 replication and VEEV nsP3 colocalizes with TFAP2A. (**A**) 293T cells were transfected with 25 or 50 nM siRNA targeting TFAP2A or eIF2S2, with an off-target negative control siRNA, DharmaFECT only treatment, or mock-transfection treatment and incubated for 72 h. Cell viability was measured, as described in Materials and Methods and calculated versus mock-transfected control. Dotted line represents the 90% cut-off point. (**B**) SiRNA knockdown and infection scheme. Then, 293T cells were transfected with 25 or 50 nM siRNA targeting TFAP2A or eIF2S2 and incubated for 72 h. Cells were infected with TC-83 for 1 h at MOI 0.1 and at 24 hpi, viral supernatants and cellular lysates were obtained, as described in Materials and Methods. Graph is representative of two independent experiments performed in triplicate (*n* = 6). Cellular lysates to confirm host protein knockdown were probed using Western blot for TFAP2A (**C**) or eIF2S2 (**D**) as described in Materials and Methods. Signal was quantified and normalized to actin loading control, calculated as fold-change vs. mock-transfected control group. Western blot and graphical data are representative of two independent experiments (*n* = 2). (**E**) Viral supernatants of siRNA transfected cells were evaluated by plaque assay, as described in Materials and Methods. Graph is representative of two independent experiments performed in triplicate (*n* = 6). Images are representative of two independent experiments. * *p* < 0.0332, ** *p* < 0.0021, *** *p* < 0.0002, **** *p* < 0.0001, and ns, not significant.

To estimate the relevance of host targets for these inhibitors in the context of alphavirus infection and siRNA knockdown experiments were carried out. The relevance of the host proteins, eukaryotic initiation factor 2 subunit 2 (eIF2S2), and transcription factor AP-2 alpha (TFAP2A) for TC-83 replication were first assessed using siRNA-mediated knockdown of the target proteins. eIF2S2 is the host protein in the immunoprecipitated pulldowns (Figure 1A–C) predicted to be targeted by tomatidine (Figure 1D). TFAP2A is predicted to be targeted by citalopram HBr and Z-VEID-FMK (Figure 1D). Next, 293T cells were transfected with 25 or 50 nM of siRNA targeting TFAP2A or eIF2S2 for 72 h and cell viability was measured to assess the toxicity of reduced host protein expression (Figure 8A). Transfection of 25 or 50 nM siRNA targeting TFAP2A and eIF2S2 did not compromise cell viability as compared to the transfection reagent control or the negative siRNA control. Cell viability was noted to be ≥94% for all conditions tested in Figure 8A. Then, 293T cells were transfected with 25 or 50 nM siRNA targeting eIF2S2 or TFAP2A for 72 h and infected with TC-83 at MOI 0.1 for 24 h (Figure 8C). Host protein knockdown was confirmed by Western blot for TFAP2A (Figure 8C) and eIF2S2 (Figure 8D), and supernatants were evaluated for infectious titers by plaque assay at 24 hpi from siRNA transfected cells (Figure 8E). Furthermore, 25 nM siRNA for TFAP2A reduced protein expression by 23% (ns) as compared to the mock-transfected cells (Figure 8C). However, 50 nM siRNA achieved a 75% knockdown (*p* < 0.0001) as compared to the mock-transfected cells, and a ~50% knockdown as compared to the negative siRNA control (Figure 8C). Transfection of 25 nM siRNA targeting eIF2S2 reduced protein expression by 55% as compared to the mock-transfected cells and the negative siRNA control (*p* < 0.0002) (Figure 8D), whereas 50 nM siRNA reduced protein expression by 76% (*p* < 0.0001) (Figure 8D). Viral supernatants, assayed for infectious particle production (Figure 8E), showed a modest reduction in TC-83 replication measured at 1.4-fold and 3.6-fold following 25 nM or 50 nM TFAP2A siRNA transfection, respectively (ns, *p* < 0.0021). However, a greater reduction in TC-83 replication was observed for the eIF2S2 knockdown (Figure 8E) in which 25 nM siRNA reduced TC-83 replication by 15-fold (*p* < 0.0002), and 50 nM reduced TC-83 replication by 74-fold (*p* < 0.0002).

**Figure 9:**
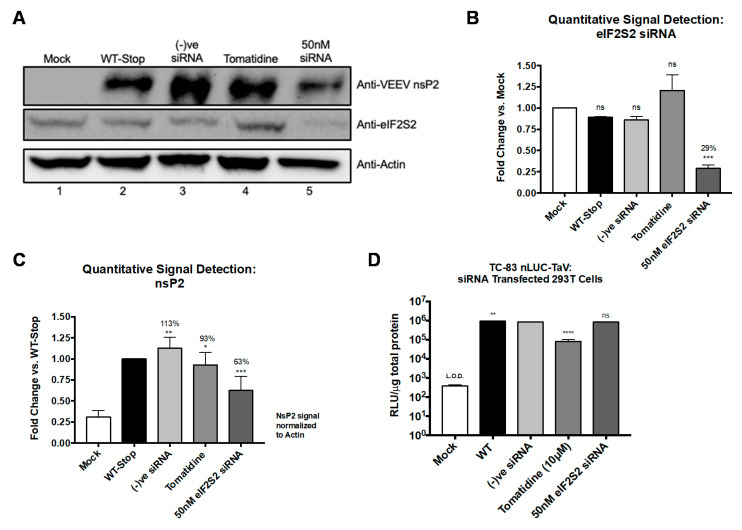
EIF2S2 is important for VEEV genomic RNA but not subgenomic RNA translation. Furthermore, 293 T cells were transfected with 50 nM eIF2S2 or off-target negative siRNA for 72 h or treated with 10 µM Tomatidine for 2 h. TC-83 genomic RNA containing a mutated stop codon was transfected, as described in Materials and Methods for 24 h. Cellular lysates were probed for eIF2S2 siRNA knockdown (**A**,**B**), nsP2 as an indicator of genomic translation (**A**,**C**), as described in Materials and Methods. Signals were quantified and normalized to the actin loading control and fold-change was calculated versus the mock-transfected cells (**B**) or the untreated and non-siRNA transfected WT-Stop control (**C**). Western blot and graphical data are representative of two independent experiments (*n* = 2). SiRNA transfected or tomatidine treated 293 T cells were infected in triplicate with a nLUC-TaV expressing TC-83 virus at MOI 0.1 for 24 h, as described in Materials and Methods. (**D**) Cellular lysates were obtained and quantified for luciferase activity and normalized to total protein by Bradford assay for measurement of subgenomic RNA translation, as described in Materials and Methods. Graph is representative of two independent experiments performed in triplicate (*n* = 6). * *p* < 0.0332, ** *p* < 0.0021, *** *p* < 0.0002, **** *p* < 0.0001, ns, not significant.

EIF2S2, a eukaryotic translation initiation factor, is the β-subunit that, along with the α- and γ- subunits, forms the eIF2 complex that initiates translation [48]. Alphavirus recruitment of eIF2 to aid in efficient genomic RNA translation has been extensively reported [8,49], whereas alphavirus subgenomic RNA translation is independent of eIF2 [50,51,52]. Further, studies have shown cell-free translation of alphavirus genomic RNA is poor but subgenomic RNA translation, without eIFs, maintained its efficiency [53]. In this context, the impact of knockdown of eIF2S2 on translational efficiency of the VEEV genome was evaluated. To test the effect of eIF2S2 knockdown on genomic RNA translation independent of replication, a mutant TC-83 genome was generated in which the leaky opal stop codon (UGA) located at the C-terminus of nsP3 was mutated to a strong ochre stop codon (UAA) in a luciferase-expressing T-83 wild-type backbone and an additional ochre stop codon was included downstream to further ensure replication abrogation (Figure S1). Next, 293 T cells were transfected with 50 nM eIF2S2 siRNA for 72 h or treated with 10 µM tomatidine for 2 h prior to transfection with 5 µg of TC-83 RNA containing the mutated stop codon or infection with the nano Luciferase expressing TC-83 virus (TaV-nLuc). Cells were lysed and processed for expression of VEEV nsP2 and eIF2S2 by Western blot (Figure 9A–C) or for luciferase expression (Figure 9D). Quantitative host protein knockdown of eIF2S2 was 71% as compared to the mock-transfected cells and ~60% as compared to the negative siRNA control (*p* < 0.0002) (Figure 9A,B). Genomic translation levels were reduced by 37% following eIF2S2 knockdown as compared to translation in the non-siRNA transfected cells (*p* < 0.0002) (Figure 9A,C). In the presence of tomatidine treatment, genomic translation was reduced by 7% as compared to the untreated cells (*p* < 0.0332) (Figure 9A,C). Detection of luciferase activity was used as a measure of subgenomic translation (TaV-nLuc) [34]. The results (Figure 9D) revealed that reduction in eIF2S2 expression reduced TC-83 subgenomic luciferase activity by ~1-fold as compared to the untreated, non-siRNA transfected control (WT) and the negative siRNA control groups (ns). Interestingly, a 12-fold decrease of luciferase activity was measured in the tomatidine-treated cells as compared to the untreated control (*p* < 0.0001). Taken together, these data suggest that eIF2S2 is important for the efficiency of VEEV genomic RNA translation, and supports previous studies indicating minimal reliance on eIF2 for subgenomic RNA translation. Furthermore, these data imply that at least some of the observed antiviral activity of tomatidine against VEEV is likely to involve an alternative host protein or pathway distinct from eIF2S2.

## 4. Discussion

VEEV, a NW alphavirus, is both a public health and a bioweapon concern as it replicates readily to high titers while retaining infectivity in the form of an aerosol. The need for broad-spectrum, cell-type-independent therapeutic intervention strategies prompted us to examine the host protein interactome of VEEV nsP3 protein. Here, we report the efficacy of three small molecules that are FDA approved for alternate indications which demonstrate potent inhibition against VEEV and appear to possess broad spectrum antiviral activity. Tomatidine displayed the greatest inhibition of VEEV replication across multiple human cell types against both the wild-type and attenuated strains of VEEV, and against the related alphavirus EEEV. Citalopram HBr and Z-VEID-FMK were efficacious against VEEV TrD as well as EEEV, therefore demonstrating broad-spectrum potential against alphaviruses. Similarly, citalopram HBr and Z-VEID-FMK were effective when used as a post-exposure treatment against VEEV. Additionally, our studies reveal a potential interaction of VEEV nsP3 with TFAP2A in the absence and presence of citalopram HBr and Z-VEID-FMK, although knockdown of TFAP2A does not negatively impact TC-83 replication. Our data suggests a proviral role of eIF2S2 for TC-83 replication and genomic translation.

Tomatidine is a steroidal alkaloid that possesses antimicrobial and anti-inflammatory properties in macrophages [54,55,56]. Previously, tomatidine has been shown to reduce iNOS and COX-2 expression by interfering with the NF-κB and JNK signaling pathways [57]. Inflammatory cytokines IL-1, IL-6, and IL-8 are stimulated during VEEV infection and these inflammatory mediators are also known to upregulate COX-2 [58,59]. To that end, non-steroidal COX-2 inhibitors, such as celecoxib, have been shown to inhibit VEEV replication in vitro while other COX inhibitors have delayed disease symptoms in vivo [60,61]. More recently, tomatidine has shown strong efficacy against the OW alphavirus, Chikungunya virus (CHIKV), as well as a flavivirus and dengue virus (DENV) [62,63]. Potent antiviral activity of tomatidine was observed against these viruses with selectivity indexes calculated at 97.7 and 120 for DENV and CHIKV, respectively, compared to 70 observed in our study [62,63]. Interestingly, the time of addition experiments with DENV showed tomatidine inhibiting viral particle production up to 12 h post-infection [62], and up to 6 h with CHIKV [63], whereas tomatidine appears to be efficacious only when added before or up to the time of infection when used against VEEV. In the case of CHIKV, direct incubation of tomatidine with the virus resulted in no inhibition of viral titer [63], which suggests that its antiviral activity likely affects steps in the replication cycle following release of the nucleocapsid but prior to budding of progeny virions [62,63]. Tomatidine disrupts eIF2S2 activity, which can affect VEEV replication as this protein appears to possess a proviral role. Eukaryotic initiation factor 2 (eIF2) forms a ternary complex with GTP to mediate the binding of tRNA_i_^Met^ to the ribosome to facilitate protein synthesis, and is comprised of three subunits: alpha (eIF2S1), beta (eIF2S2), and gamma (eIF2S3) [48]. In the case of OW alphavirus, SINV, subgenomic RNA translation is independent of eIF2 activity [51,52]. A stable RNA hairpin motif present slightly downstream to the AUG start codon in the subgenomic RNA is conserved among OW and NW alphaviruses, and in SINV, is responsible for the eIF2-independent translation [49,50,64]. Our data demonstrate that eIF2S2 aids in translation of the viral genomic RNA, but not the subgenomic RNA, similarly to SINV [49,50,51,52]. Interestingly, genomic RNA translation was modestly affected by treatment with tomatidine, but subgenomic RNA translation was significantly reduced. Nevertheless, our bioinformatics analyses successfully provided a two-fold identification of a small molecule inhibitor and a host target of interest with VEEV nsP3.

Depletion of TFAP2A had little to no impact on VEEV replication, which is unsurprising given that the full-length genome encodes its own proteins for transcribing the negative-, positive- and subgenomic-RNA strands, and thus a direct role for TFAP2A in viral RNA synthesis appears unlikely. However, the inhibitors identified as putatively targeting TFAP2A displayed efficacy against VEEV, which may suggest an indirect role for TFAP2A in the VEEV replication cycle, or that the antiviral activity of these inhibitors is due to off-target effects on proteins/pathways other than TFAP2A. Caspase-induced apoptosis occurs during alphavirus infection, and in the case of SINV and Semliki Forest virus (SFV), caspase-3 is activated by viral cleavage of the Bcl-2 family of oncogenes to prevent host cell death, and is used as a mechanism of protection [65,66]. However, treatment with a different caspase-3 inhibitor, Z-VAD-FMK, reduced SINV-induced cellular death but did not significantly affect viral particle production in BHK-J cells [66]. In another study, Bcl-2 cleavage was blocked during treatment with Z-VAD-FMK prior to SFV infection, thereby preventing apoptosis. However, it is unclear whether reduction in cell death is due to lack of caspase-3 activation or intact Bcl-2. Similarly, Z-VAD-FMK did not reduce the translation of viral proteins or infectious particle production [65,67]. Our data support further investigation into the mechanism by which Z-VEID-FMK reduces infectious particle production. Relatedly, the FDA-approved SSRI, citalopram HBr, typically used to treat depression, was also shown to decrease apoptosis in osteoblast cells in vitro [45,68]. Research investigating potential antiviral activity mediated by citalopram HBr against alphaviruses is lacking, but ex vivo studies have demonstrated that treatment with the SSRI decreased human immunodeficiency virus (HIV) replication in both macrophages and in latently infected T-cells [69]. Taken together, these two small molecules display comparable efficacy against VEEV during viral kinetic and post-addition assays and therefore, may target related or similar host pathways to negatively affect viral propagation.

## 5. Conclusions

Overall, we have presented in vitro evidence demonstrating the antiviral activity of three re-purposed small molecule inhibitors against NW alphaviruses. Our data suggest that utilizing overexpression constructs expressing viral proteins combined with mass spectrometry and bioinformatic analyses can identify host interaction partners and small molecules of interest that may be repurposed for novel uses. Future studies will be aimed toward defining the mechanistic basis for the antiviral activity of these small molecules and determining their efficacy against alphaviruses in vivo.

## Data Availability

Data is available in Supplementary Figure S1 and Mass spectrometry data is available in Supplementary Tables S1 and S2. All other data is available upon request.

## Supplementary Materials

The following are available online at https://www.mdpi.com/article/10.3390/v13081533/s1, Figure S1: Site-directed mutagenesis. Figure S2: Nsp3-HA confirmation, Table S1: Host interactors, Table S2: Identified inhibitors.
