# Supplementary material for: Inhibitors of Venezuelan Equine Encephalitis Virus Identified Based on Host Interaction Partners of Viral Non-Structural Protein 3
Source: Viruses. 2021 Aug 3;13(8):1533. doi: 10.3390/v13081533 (PMC8402862; doi:10.3390/v13081533)
Supplement: Supplementary file 1 [file viruses-13-01533-s001.zip › Supplementary Figure File - mol.pdf]

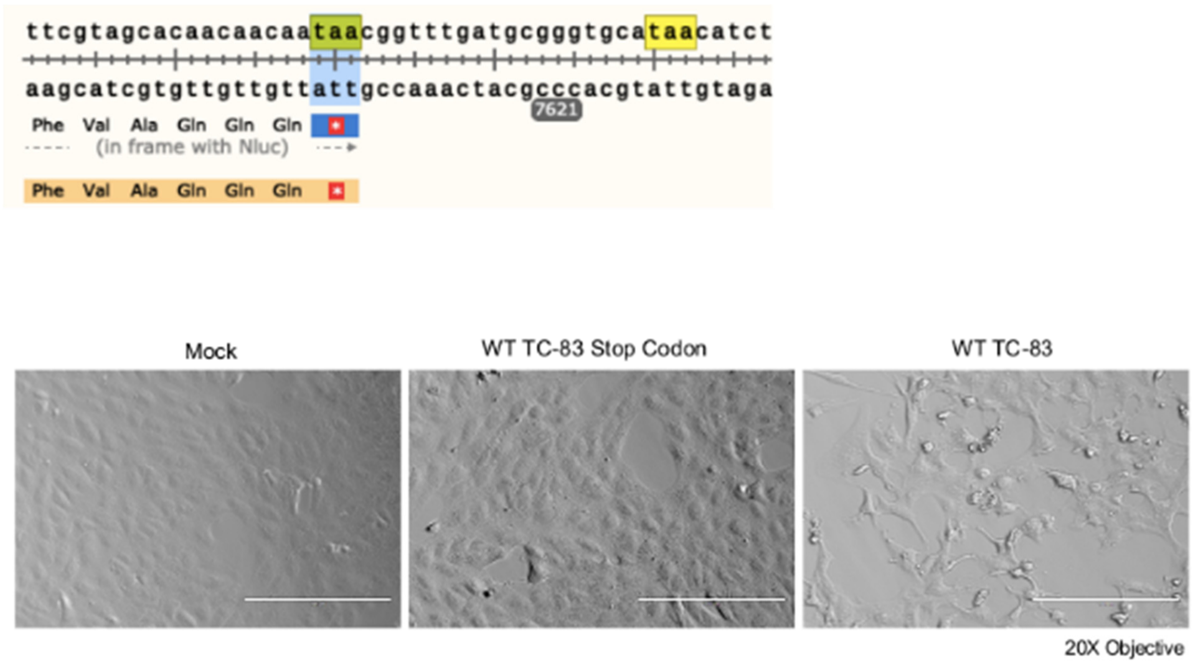

Figure S1. Site-directed mutagenesis of the opal stop codon (UGA) in the TC-83 nLUC-TaV virus to an ochre stop codon (UAA) was generated to abrogate replication ability. An additional ochre stop codon was placed downstream of the nsP3 opal site to in case of a read-through. Validation of replication abrogation (middle panel) was evaluated by cytopathic effects of electroporated RNA in Vero cells compared to mock-electroporated cells (left panel) and the non-mutated TC-83 electroporated cells (right panel).

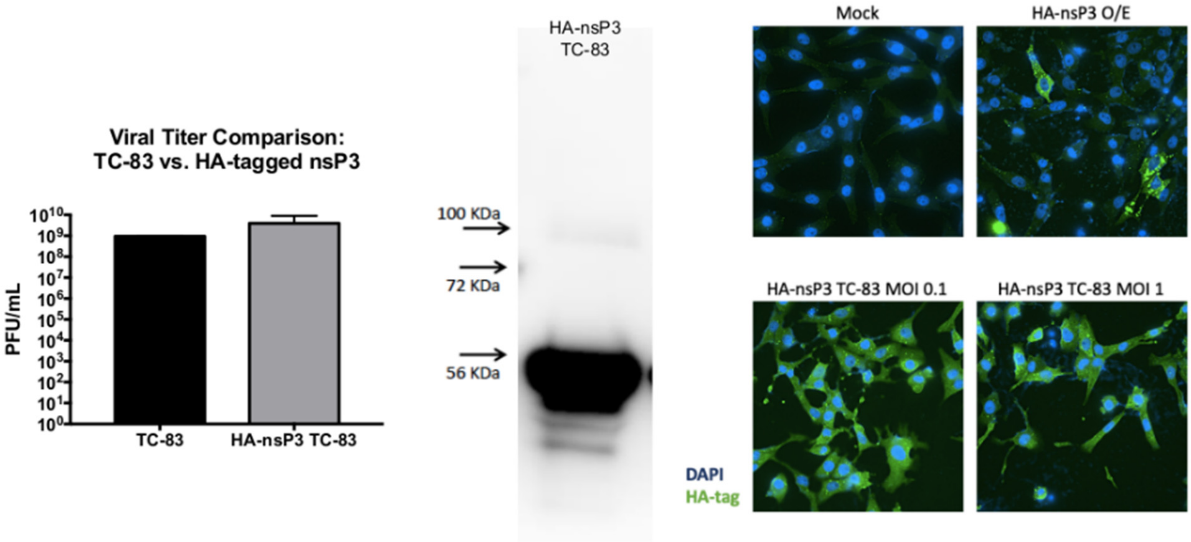

Figure S2. The nsP3-HA-tagged TC-83 nLUC TaV virus was generated by site-directed mutagenesis. HA-tag expression was confirmed by western blot (middle panel) and confocal

microscopy (right panel) in comparison to an overexpression plasmid for nsP3-HA-tag. Interference of the HA-tag on viral replication was evaluated by plaque assay in comparison to TC-83 replication (left panel).

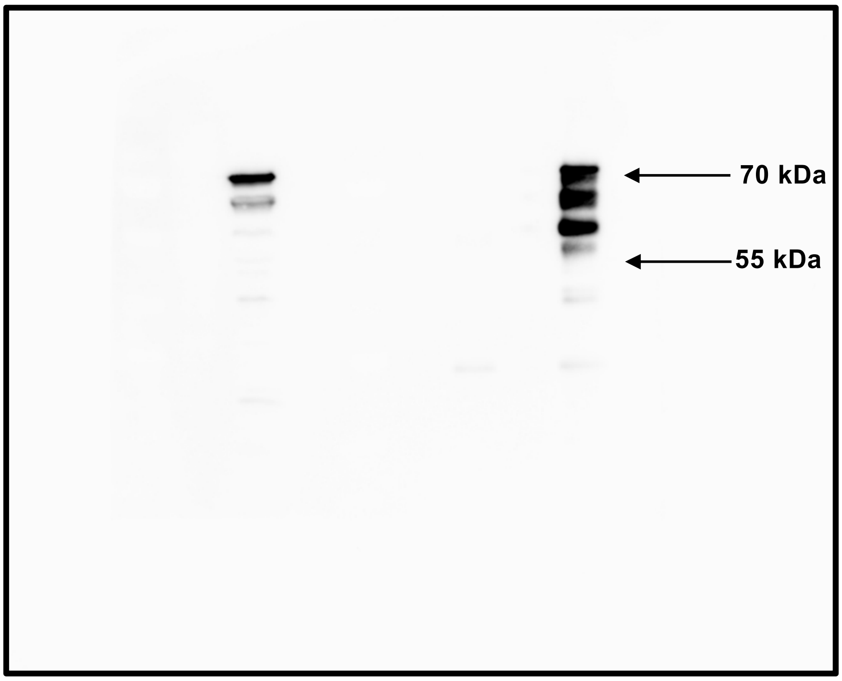

Full membrane of Immunoprecipitated nsP3-HA-tag blot. Anticipated weight: ~65kDa.

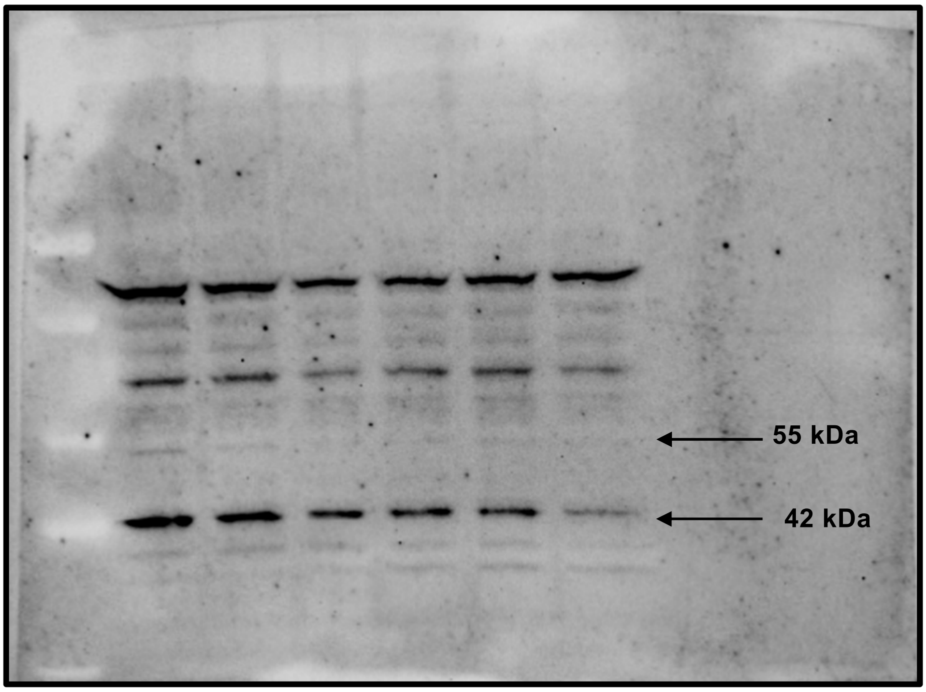

Full membrane of TFAP2A siRNA blot. Anticipated weight: 48kDa.

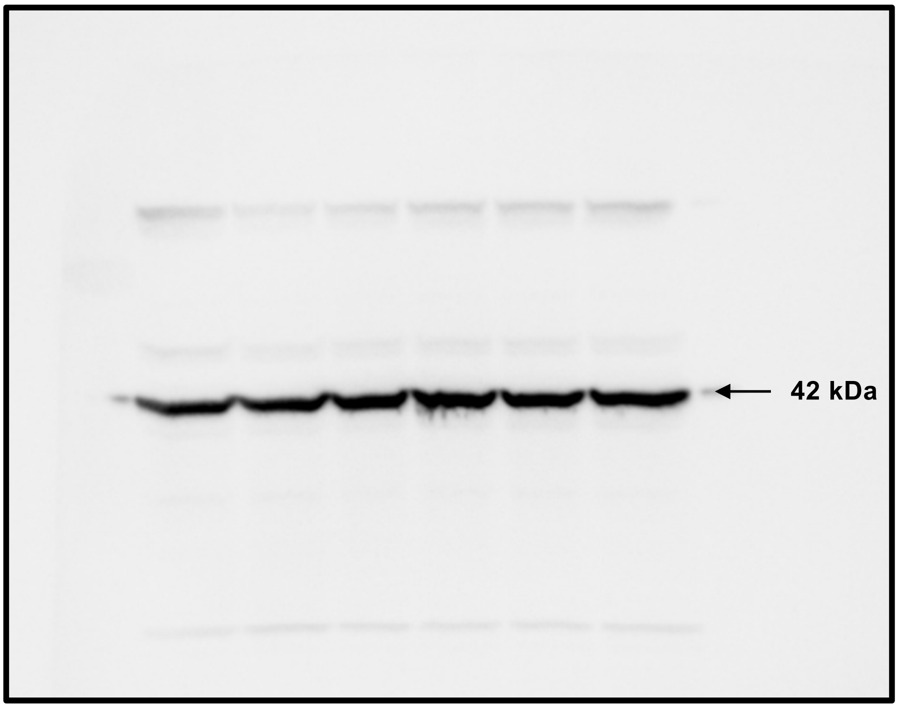

Full membrane of Actin loading control for TFAP2A siRNA blot. Anticipated weight: 42kDa

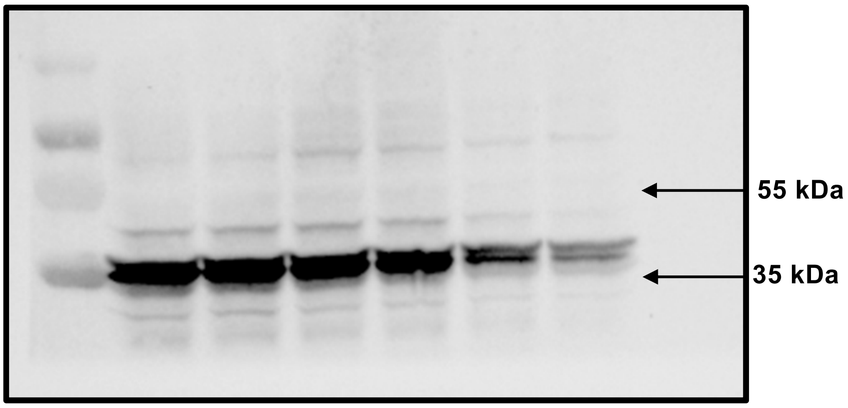

Full membrane of eIF2S2 siRNA blot. Anticipated weight: 38kDa

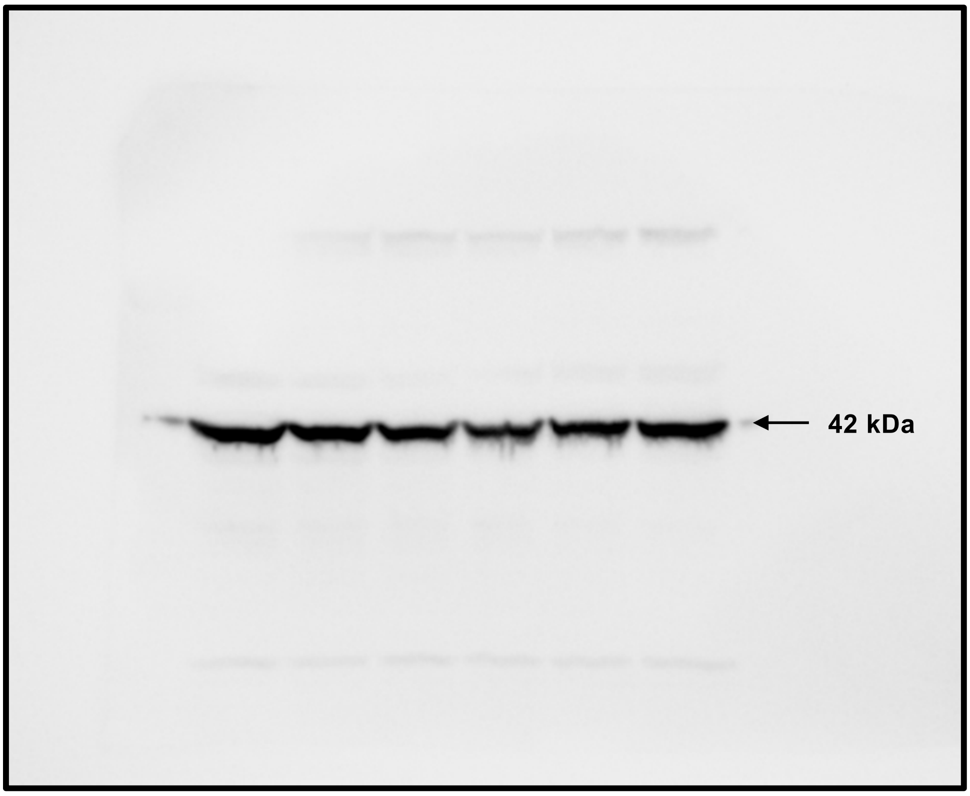

Full membrane of Actin loading control for eIF2S2 siRNA blot. Anticipated weight: 42kDa.

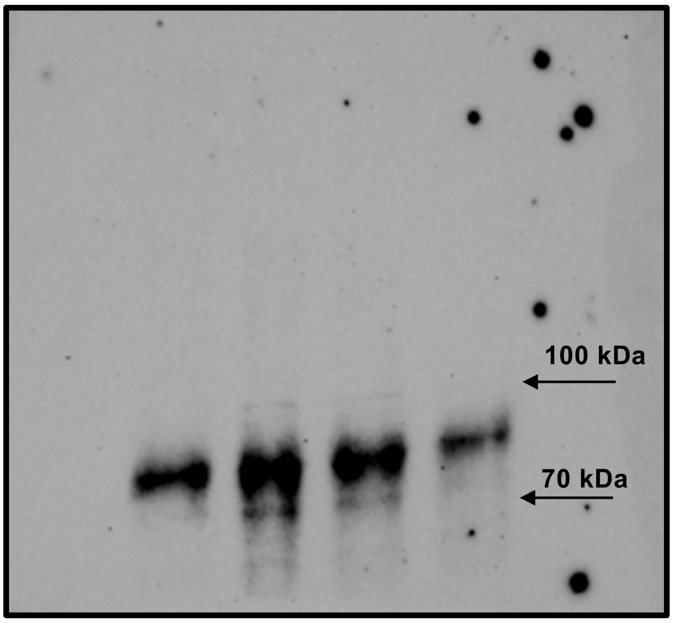

Full membrane of nsP2 blot. Anticipated weight: 90kDa.

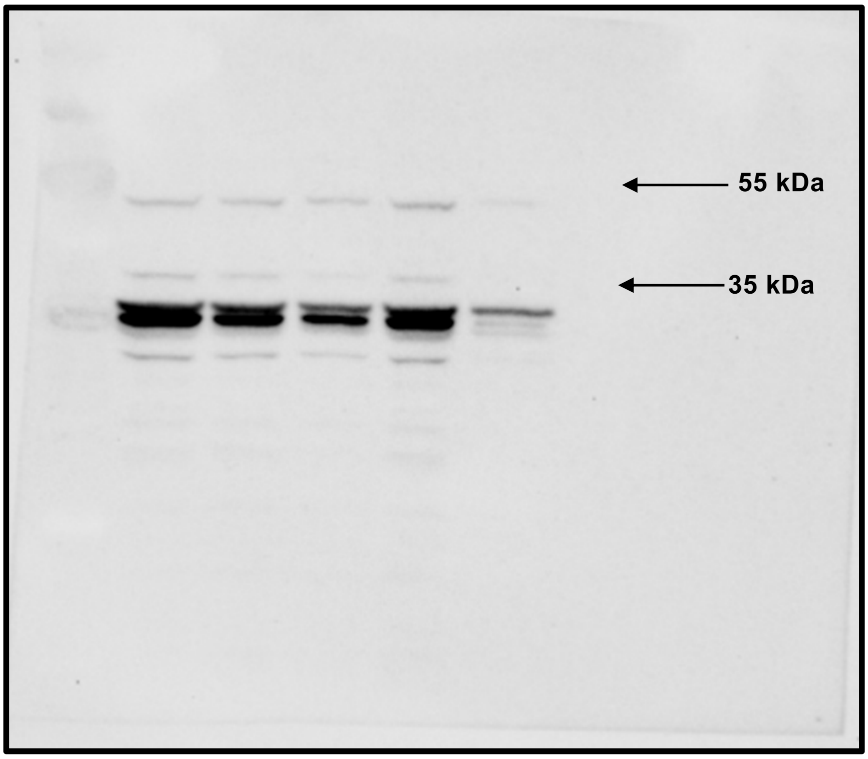

Full membrane of eIF2S2 siRNA for nsP2 blot. Anticipated weight: 38kDa.

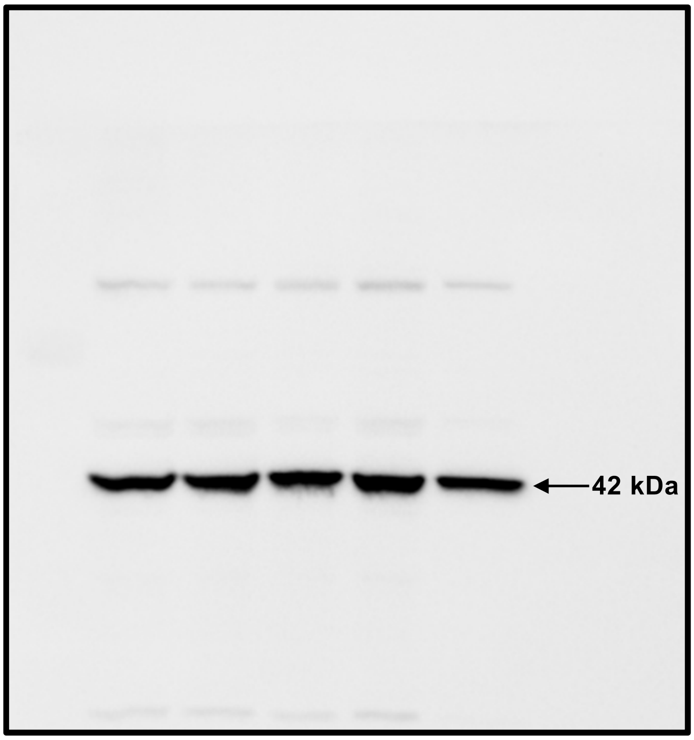

Full membrane of Actin loading control for nsP2 blot. Anticipated weight: 42kDa.
